# Integrating Multiple Single-Cell RNA Sequencing Datasets Using Adversarial Autoencoders

**DOI:** 10.3390/ijms24065502

**Published:** 2023-03-13

**Authors:** Xun Wang, Chaogang Zhang, Lulu Wang, Pan Zheng

**Affiliations:** 1 College of Computer Science and Technology, China University of Petroleum East China, Qingdao 266580, China; wangsyun@upc.edu.cn (X.W.);; 2 Department of Accounting and Information Systems, University of Canterbury, Christchurch 8041, New Zealand

**Keywords:** scRNA-seq, batch effect, deep learning, adversarial autoencoders

## Abstract

Single-cell RNA sequencing (RNA-seq) has been demonstrated to be a proven method for quantifying gene-expression heterogeneity and providing insight into the transcriptome at the single-cell level. When combining multiple single-cell transcriptome datasets for analysis, it is common to first correct the batch effect. Most of the state-of-the-art processing methods are unsupervised, i.e., they do not utilize single-cell cluster labeling information, which could improve the performance of batch correction methods, especially in the case of multiple cell types. To better utilize known labels for complex dataset scenarios, we propose a novel deep learning model named IMAAE (i.e., integrating multiple single-cell datasets via an adversarial autoencoder) to correct the batch effects. After conducting experiments with various dataset scenarios, the results show that IMAAE outperforms existing methods for both qualitative measures and quantitative evaluation. In addition, IMAAE is able to retain both corrected dimension reduction data and corrected gene expression data. These features make it a potential new option for large-scale single-cell gene expression data analysis.

## 1. Introduction

The rapid development of high-throughput single-cell RNA sequencing (scRNA-seq) technologies has facilitated the study of the transcriptomic characterization of cell heterogeneity and dynamics [1,2,3,4]. In recent years, researchers have collected a large amount of single-cell gene expression data from different experiments at different times and on different sequencing platforms [5,6]. Inevitably, these data will have unexpected batch effects due to differences in time and experimental protocols, which may lead to spurious findings [7]. Therefore, correcting the batch effect should be an essential part of the analysis of multi-batch scRNA-seq data.

At present, researchers have proposed a number of methods for batch effect correction [8]. However, almost all of these methods are unsupervised, i.e., they do not use cell-type information, including cell similarity-based methods such as MNN [9], BBKNN [10], Scanorama [11], clustering-based methods such as Harmony [12], DESC [13], a low-rank subspace ensemble framework [14], SCCLRR [15], and a novel strategy based on Autoencoders [16]. Although all of these methods have achieved some results, their effectiveness may vary depending on the complexity of the dataset. Therefore, it is important to carefully consider which method to use for a particular dataset, taking into account its unique characteristics and limitations.

**Figure 1:**
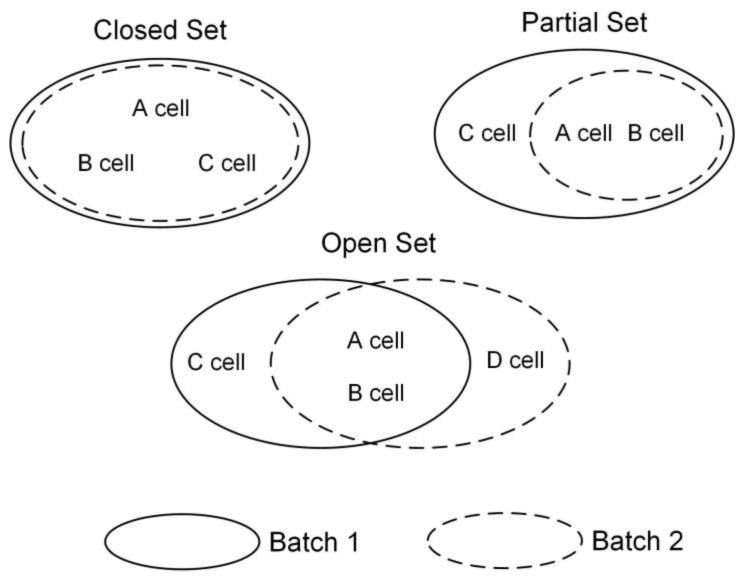
Closed set: each batch has the same type of cells. Partial set: the set of cell types in one batch is a subset of those in another batch. Open set: each batch contains both the same and different types of cells. Only two batches are illustrated; multiple batches can be analogous to the example.

After an in-depth analysis of the scRNA-seq datasets, we identified three different scenarios (Figure 1): Closed set, where each batch contains exactly the same cell type; partial set, where the set of cell types in one batch is a subset of those in another batch; and open set, where each batch contains both the same and different types of cells, making it the most complex and realistic situation that current unsupervised methods cannot effectively resolve.

Fortunately, the development of single-cell study and annotation methods is rapidly advancing [17,18,19]. As a result, an increasing number of publicly available annotated single-cell datasets [8] are now available, making it easier to capture information on cell types. Building on this progress, we developed a supervised method IMAAE to correct the batch effect in the above three scenarios. IMAAE utilizes cell type information to establish associations of the same type of cells between different batches. The method can either build a new batch or select one of the existing batches as an anchor and then use an adversarial autoencoder to convert the remaining batches to the anchor batch, effectively correcting the batch effect.

We compare IMAAE with a variety of advanced batch correction methods, including the most widely used MNN, the more recent iMAP [20], and SCALEX [21] based on deep learning techniques and a supervised method, scGEN [22]. Experimentally, our method proved to be better than other methods in the standard set of evaluation metrics. IMAAE can obtain both corrected low-dimensional data and corrected gene expression data, providing strong support for downstream analysis.

## 2. Materials and Methods

**Figure 2:**
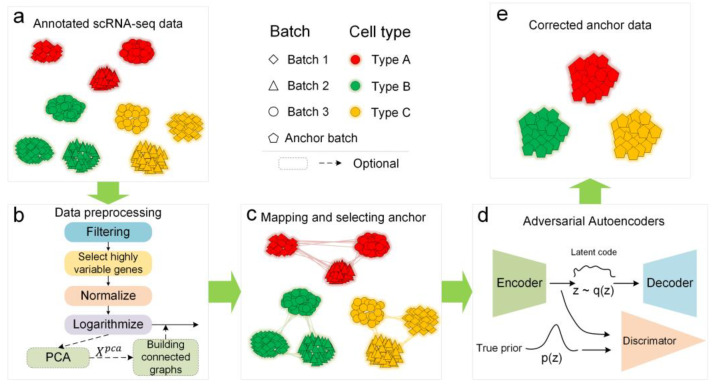
The overall description of the proposed IMAAE framework. (**a**) Get the annotated scRNA-seq dataset. (**b**) Data preprocessing. (**c**) Establish mapping relationships for different batches of the same type of cells and select the anchor batch. (**d**) The preprocessed data is fed to the adversarial autoencoder for training according to the mapping relationship. (**e**) Batch effects are corrected using a trained adversarial autoencoder.

Our IMAAE framework workflow includes 3 major phases. There are two tasks in the first phase, data annotation, and processing. It aims to produce normalized data and neighbor connectivity maps after initial denoising (Figure 2a,b). The second phase is the anchor selection phase, where a certain batch is selected as the anchor batch, or an intermediate batch is established as the anchor batch (Figure 2c). The third phase is the batch effect correction phase (Figure 2d,e), where, based on the established mapping relationships, all batches of cells as the input data of the antagonistic autoencoder, and the anchor batch cells as the ideal output data for training, and then the batch effect can be corrected by the trained network. IMAAE is available at https://github.com/dongzuoyk/IMAAE (last access date: 9 March 2023).

### 2.1. Data Preparation and Data Preprocessing

The data used in this study are publicly available annotated datasets.

Human peripheral blood mononuclear cell dataset (PBMC) [23]. The data included two batches of human peripheral blood mononuclear cells from two healthy donors, which were generated by the 3′and 5′Genomics protocols, respectively. Each batch contained 12 different cell types, including 8098 cells in the 3′ batch and 7378 cells in the 5′ batch, with 33,694 genes per cell.

Human pancreas dataset (Pancreas) [24,25,26,27,28]. This dataset was constructed using human pancreatic data from five different sources. Each batch contained 15 different cell types, for a total of 14,890 cells with 34,363 genes per cell.

The data preprocessing step includes (Figure 2b): (1) Filtering, i.e., removing unwanted cells and genes according to user-defined rules. Cells expressing less than 600 genes and genes expressed in less than 3 cells were excluded from this study. (2) Selecting highly variable genes. Since the original gene dimension is very high and contains a large number of zero values, the study should focus on those high-variable genes. In this study, 2000 high-variable genes were selected for the study. (3) Normalization. Each cell was normalized by the total counts of all genes so that each cell had the same total count after normalization. The target total count for this study was 20,000. (4) Logarithmization. To logarithmize the gene expression data, in this paper, we used X = log(X + 1). (5) Principal component analysis (PCA). PCA was performed on the logarithmically scaled data to obtain the reduced dimensional data for constructing similar cell connectivity graphs. (6) Building connected graphs. Constructing connectivity graphs between cells of identical types across distinct batches. The cells on the connected graph are highly similar, and the spatial distance is smaller than other cells of the same type across batches. Note that steps (5) and (6) are the procedures for constructing the cross-batch similarity cell connected graph in the IMGG model [29], which are optional in the IMAAE model. The difference is that if these steps are not used, random selection will be adopted when establishing mappings between different batches of cells of the same type in subsequent steps. This method is fast and convenient, and the corrected data distribution will be more uniform, but the ability to identify cell subtypes will be lost. In contrast, if steps (5) and (6) are used, cells on the connected graph will be selected when establishing mappings between different batches of cells of the same type in subsequent steps, and the corrected data will still retain some batch-specific features that can theoretically be used for subtype analysis. We further elaborate on this in Additional Experiment 2.

### 2.2. Determining the Anchor Batch

IMAAE is a flexible anchor-based method. Unlike other anchor-based methods, IMAAE can not only choose a certain batch as the anchor batch but likewise choose to construct an intermediate batch as the anchor batch, such as IMGG.

In this work, we provide three ways to select an anchor batch:

(1) Similar to IMMG, an intermediate batch is established as an anchor batch using the balanced mode.
(2) A batch with a larger standard deviation is selected as the anchor batch. A larger standard deviation means that there is greater variability in the cells within the batch, which may cover more cell types.
(3) The user can choose a batch as the anchor batch themselves.

Different methods of establishing the anchor batch result in slightly different outcomes, but experimental results show that regardless of the method used, the IMAAE correction effect is always excellent (Additional Experiment 3). Unless otherwise specified, in this paper, the intermediate batch established using the balanced mode is used as the anchor batch for all other experiments.

### 2.3. Correcting the Batch Effect Using an Adversarial Autoencoder

Our purpose is to transform all batches of cells to the anchor batch to correct the batch effect. Converting one batch to another is similar to style migration in the image domain, and the methods generally used are autoencoder and generative adversarial networks. We chose to design an adversarial autoencoder because autoencoder and generative adversarial networks have their own limitations in dealing with complex dataset scenarios (Additional Experiment 1).

#### 2.3.1. Adversarial Autoencoder Network

To address the limitations of the autoencoder and generative adversarial networks, we decided to design an adversarial autoencoder network by fusing the structures of the autoencoder and generative adversarial networks (Figure 2d). Our model contains three parts: encoder, decoder, and discriminator. The encoder and decoder can form a self-encoder network, and the encoder and discriminator can form a generative adversarial network.

First, we input all batches of gene expression data *x* into the encoder to obtain the latent code *z*, which is distributed as *q*(*z*). Next, *z* is fed to the decoder for training to obtain data matching the features of the anchor batch, while the encoder and discriminator form a generative adversarial network to match the distribution *q*(*z*) of *z* with the true prior distribution *p*(*z*). Eventually, IMAAE can learn the parameters for converting all batches into anchor batches. In our work, we used the normal Gaussian distribution *N*(0, *I*) for the prior distribution *p*(*z*).

#### 2.3.2. Loss Functions

The reconstruction loss function of the autoencoder:

(1) $$L_{R}=\frac{1}{n}\overset{n}{\underset{i=1}{\sum }}{\left(x_{i}-{\tilde{x}}_{i}\right)}^{2}$$

where n denotes the number of cells in the dataset, $x_{i}$ denotes the original gene expression of the *i*-th cell, and ${\tilde{x}}_{i}$ denotes the gene expression generated by the autoencoder for cell i.

The discriminant loss function for generative adversarial networks:

(2) $$L_{D}=-E_{z∼p_{data}\left(z\right)}\left[D\left(z\right)\right]+E_{z^{\prime }∼p\left(z^{\prime }\right)}[D\left(z^{\prime }\right)+\lambda E_{\hat{z}∼p_{\hat{z}}\left(\hat{z}\right)}[{\left(\|\nabla_{\hat{z}}D\left(\hat{z}\right){\|}_{2}-1\right)}^{2}]$$

where z is the true sample, $p_{data}\left(z\right)\;$ is the true data distribution, $\hat{z}$ is the interpolated sample obtained by sampling uniformly between the true sample z and the noisy sample $z^{\prime }$, λ is the weight of the gradient penalty term, $\|\cdot {\|}_{2}$ is the $L_{2}$ parametrization, and $\nabla_{\hat{z}}D\left(\hat{z}\right)$ is the gradient of the discriminator at $\hat{z}$.

$p_{\;\hat{z}\;}\left(\;\hat{z}\;\right)$ is the distribution of the interpolated samples, defined as:

(3) $$p_{\hat{z}}\left(\hat{z}\right)=E_{\alpha ∼U\left(0,1\right)}\left[\delta \left(\hat{z}-\alpha z+\left(1-\alpha \right)z^{\prime }\right)\right]$$

where $U\left(0,1\right)\;$ is a uniform distribution over the interval $\left(0,1\right)\;$ and δ  is a Dirac delta function.

The generator loss function for generative adversarial networks:

(4) $$L_{G}=-E_{z^{\prime }∼p\left(z^{\prime }\right)}\left[D\left(z^{\prime }\right)\right]$$

where $z^{\prime }$ is the noise sampled from the prior distribution $p\left(z^{\prime }\right)$, $D\left(\cdot \right)$ is the discriminator function, and E is the expectation operator.

#### 2.3.3. Hyperparameters

We used the Adam optimizer [30] with parameters β1 = 0.5 and β2 = 0.999, a learning rate of 0.0002, a batch size of 1024, and an epoch number of 100. The encoder, decoder, and discriminator all consisted of a fully connected neural network. By default, the number of encoder nodes is 2000, 1000, 500, and 250, the number of decoder nodes is 250, 500, 1000, and 2000, and the number of discriminator nodes is 250, 125, 64, 8, and 1. It should be noted that the ReLU activation function [31] must be used at the end of the decoder, which ensures that the output will not have negative numbers. We also use the reparameterization technique at the end of the encoder. In addition, we have a hyperparameter “n_critic” to adjust the training ratio between the generative adversarial network and the self-encoder, e.g., when set to 2, the generative adversarial network will be trained twice and the self-encoder once. We recommend keeping the size of “n_critic” the same as the number of batches.

### 2.4. Comparison Methods

This study compared the performance of batch correction using scGen, MNN, iMAP, SCALEX, and Harmony methods, respectively. The first three methods can only obtain corrected gene expression data, Harmony can only obtain corrected low-dimensional data, and SCALEX, similar to our IMAAE, can obtain both corrected low-dimensional data and corrected gene expression data. We used the same data preprocessing method for all methods (except SCALEX, which has an integrated preprocessing module). For each method, we run with default parameters. To assess the performance of each method, including IMAAE, the top 50 PC vectors extracted from the batch-corrected expression matrix were used for the calculation of evaluation metrics and visualization.

### 2.5. Evaluation Metrics

To evaluate the batch correction performance of IMAAE and the other methods described above, we used three quantitative assessment metrics, average silhouette width (ASW) score [32], adjusted rand index (ARI) score [33], and normalized mutual information (NMI) score [34], and two qualitative assessment metric, uniform manifold approximation and projection (UMAP) visualization [35] and t-distributed stochastic neighbor embedding (t-SNE) visualization [36]. The UMAP plot and t-SNE plot can visualize the change before and after correcting the batch effect. ASW, ARI, and NMI are metrics used to evaluate clustering quality. ASW measures the similarity of data points within a cluster to those in other clusters and evaluates cluster separation and compactness. ARI measures clustering similarity while accounting for chance agreement, often used for comparing ground truth and clustering results. NMI measures mutual information between two clustering results and is normalized by entropy. Lower ASW, ARI, and NMI scores indicate better results when using batch as a label, while higher scores indicate better results when using cell type as a label. For comparison purposes, we calculated the *F*1 score for each metric (e.g., $F1_{ASW}=\frac{2\times \left(1-ASW_{batch}\right)\times ASW_{celltype}}{1-ASW_{batch}+ASW_{celltype}}$), so that higher values indicate better performance. All scoring metrics were calculated only for the cell types that were co-occurring in each batch.

## 3. Results

We simulated three different scenarios using the PBMC dataset, the Pancreas dataset, and their subsets, i.e., closed set, partial set, and open set, respectively.

**Figure 3:**
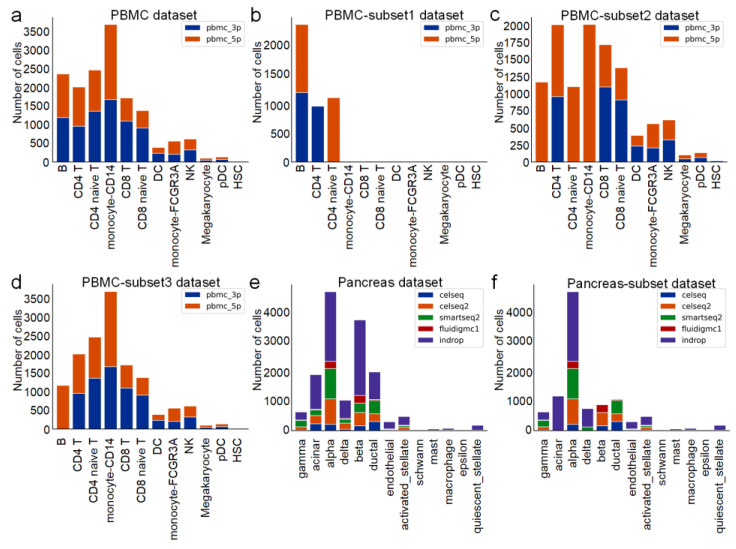
The number of cells in different batches for each dataset. (**a**) PBMC dataset, (**b**) PBMC-subset1 dataset, (**c**) PBMC-subset2 dataset, (**d**) PBMC-subset3 dataset, (**e**) Pancreas dataset, (**f**) Pancreas-subset dataset.

The closed set scenario has the PBMC dataset (Figure 3a), Pancreas dataset (Figure 3e); the partial set scenario has the PBMC-subset2 dataset (Figure 3c, compared to the PBMC dataset, we removed B cells, CD4 T cells, and monocyte-CD14 cells in 3p batch), PBMC-subset3 dataset (Figure 3d, compared to PBMC dataset, we removed B cells and CD4 cells in B cells in the 3p batch); the open set scenario has the PBMC-subset1 dataset (Figure 3b, which consists of B cells and CD4 cells in the 3p batch and CD4 cells and CD4 naïve T cells in the 5p batch of the PBMC dataset) and the Pancreas-subset dataset (Figure 3f, compared to the Pancreas dataset, we removed ‘ductal’ and ‘beta’ cells in the indrop batch, acinar and beta cells in the smartseq2 batch, acinar and delta cells in the celseq2 batch, acinar and delta cells in the celseq batch, and acinar and delta cells in the fluidigmc1 batch).

### 3.1. IMAAE Performance for the Closed Set Scenarios

We first conducted experiments on the Pancreas and PBMC datasets to evaluate the performance of the batch correction method in closed-set scenarios.

**Figure 4:**
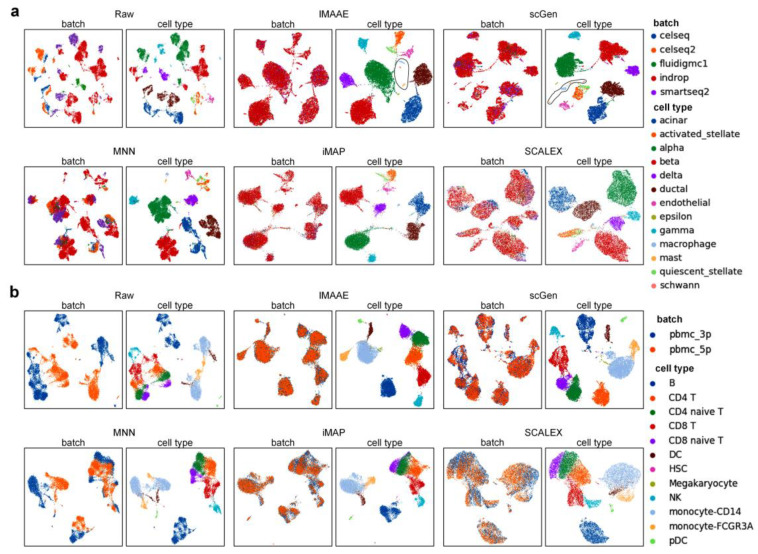
Qualitative evaluation of each batch correction method using UMAP visualization on (**a**) the Pancreas dataset and (**b**) the PBMC dataset. Each method contains two UMAP plots. On the left, coloring by batch type, and on the right, coloring by cell type.

**Figure 5:**
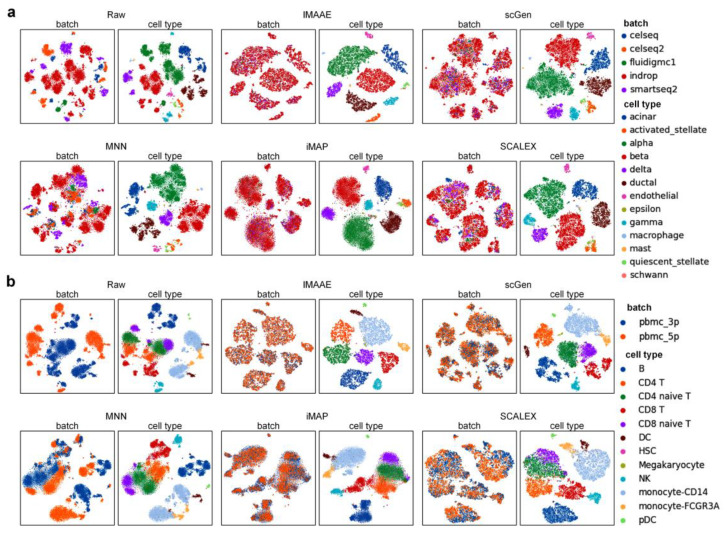
Qualitative evaluation of each batch correction method using t-SNE visualization on (**a**) Pancreas dataset and (**b**) PBMC dataset. Each method contains two T-SNE plots. On the left, coloring by batch type, and on the right, coloring by cell type.

On the Pancreas dataset and PBMC dataset, the first qualitative assessment was performed, and both UMAP visualization (Figure 4) and t-SNE visualization (Figure 5) showed that the original data had a large batch effect, while after correction by IMAAE, scGen, iMAP, and SCALEX, the batch effect has largely disappeared. On the Pancreas dataset UMAP plots show that IMAAE can discriminate some cell clusters with low numbers, and on the PBMC dataset t-SNE plots show clearer boundaries of different types of cell clusters compared to other methods IMAAE. The least effective method is MNN, which can only reduce the batch effect but cannot mix different batches of the same type of cells. Then quantitative assessment was performed (Table 1), and IMAAE had the highest *F*1 scores for all three assessment metrics and also obtained the highest scores in terms of cell types and substantially outperformed the other methods, with no significant difference between IMAAE and the other methods in terms of mixing different batches (the difference in scores was less than 0.1).

The above two experiments show that all methods except the MNN method can effectively deal with the closed set problem. At the same time, our IMAAE performs optimally on each evaluation metric. We also note that these three quantitative evaluation methods cannot effectively assess the degree of batch mixing (the difference in scores for each method is less than 0.1 and does not match the UMAP visualization plot).

### 3.2. IMAAE Performance for the Partial Set Scenarios

We carried out experiments on the PBMC-subset2 dataset to evaluate the performance of the batch correction method in partial set scenarios.

**Figure 6:**
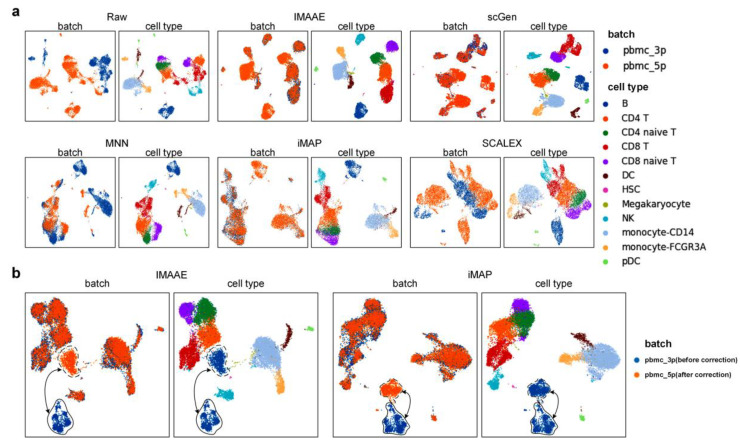
(**a**) Qualitative evaluation of each batch correction method using UMAP visualization on PBMC-subset2 dataset. (**b**) Mismapping phenomenon of PBMC-subset3 dataset before and after correction. Each method contains two UMAP plots. On the left, coloring by batch type, and on the right, coloring by cell type.

On the PBMC-subset2 dataset, the qualitative evaluation was first performed, and the UMAP visualization plot (Figure 6a) showed that the original data had a large batch effect. Meanwhile, after correction by IMAAE, scGen, and iMAP, the batch effect had largely disappeared, and IMAAE was better than other methods in distinguishing different cell types, while SCALEX did not achieve the desired effect. We speculated that SCALEX was not applicable to the scenario, and the MNN method was similarly unsatisfactory. Then the quantitative assessment was performed (Table 1), and IMAAE had the highest *F*1 scores for all three assessment metrics.

At the same time, we identified an often-overlooked problem, where if a certain type of cell appears in only one batch, improper processing will result in mapping to a nonexistent space, and the corrected data will be questioned. We used the PBMC-subset3 dataset for illustration, where B cells only appear in the pbmc_5p batch. We compared only the iMAP and IMAAE methods because the MNN effect was too poor, and the corrected data from SCALEX and scGen did not match the true gene expression data. Since B cells are present in only one batch, the usual practice is to correct B cells with the help of corrected parameters obtained using other cells using the same parameters. We expect to correct the batch effect of other cells while making B cells corrected as well. However, by converting pbmc_5p to pbmc_3p and then comparing the corrected pbmc_5p data with the uncensored pbmc_3p data, we found that the pbmc_5p batch of B cells did not overlap with the pbmc_3p batch of B cells (Figure 6b) so that whether the corrected B cells are still biologically meaningful would be questioned. Therefore, in IMAAE, we did not convert the cells that appeared in only one batch but kept the status quo.

### 3.3. IMAAE Performance for the Open Set Scenarios

Experiments were also implemented on the PBMC-subset1 dataset to evaluate the performance of the batch correction method in an open-set scenario.

**Figure 7:**
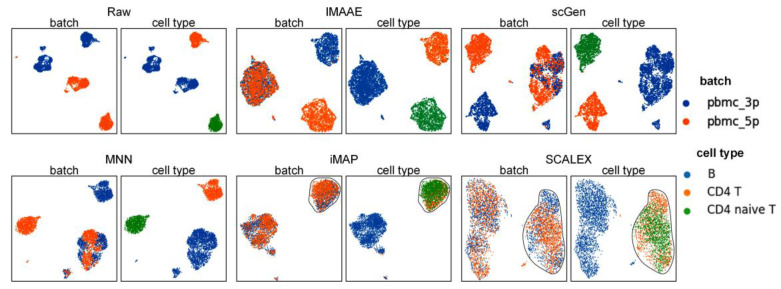
Qualitative evaluation of each batch correction method using UMAP visualization on PBMC-subset1 dataset. Each method contains two UMAP plots. On the left, coloring by batch type, and on the right, coloring by cell type.

On the PBMC-subset1 dataset, the first qualitative assessment was performed. The UMAP visualization plot (Figure 7) showed that the raw data had a large batch effect. IMAAE could mix different batches and keep different cell types separated, iMAP and SCALEX incorrectly mixed CD4 T cells with CD4 naive T cells, and scGen and MNN were less effective. Since we can already judge the performance of the method by UMAP plots alone, no quantitative analysis was performed in this experiment. It is worth mentioning that this dataset was carefully designed by us, CD4T cells and CD4 naive T cells have large similarities, and the similarity is greater than a certain degree. Almost all existing unsupervised methods failed, and only supervised methods can be used to deal with it.

### 3.4. IMAAE Performance on Low-Dimensional Data and Gene Expression Data

Another advantage of IMAAE over other methods is that it is easy to obtain corrected data in low-dimensional space and gene expression data because of the adversarial autoencoder structure.

**Figure 8:**
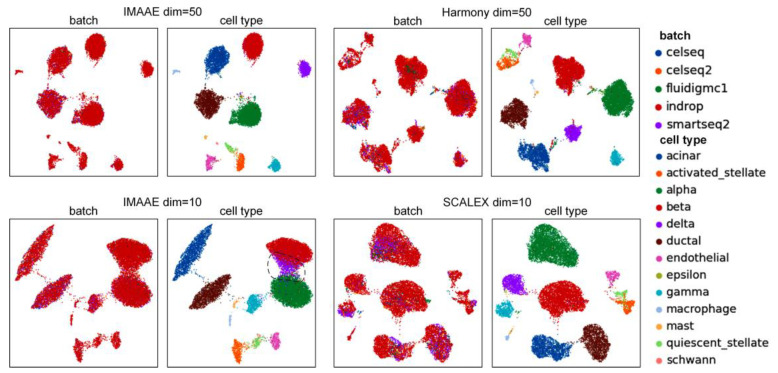
UMAP visualization plots of IMAAE, Harmony, and SCALEX corrected low-dimensional data on the Pancreas dataset.

Harmony and SCALEX can also obtain corrected low-dimensional data, but they have limitations in terms of dimensionality setting. Harmony can only obtain 50-dimensional data, and SCALEX can only obtain 10-dimensional data. In contrast, IMAAE is more flexible in this aspect, allowing researchers to select low-dimensional data based on different needs and preserve maximum original data information for downstream analysis tasks. We conducted experiments on the Pancreas dataset, and the UMAP plot shows (Figure 8) that IMAAE performs well at dimension 50, and the batch mixing effect is better than Harmony. At dimension 10, the delta cells of IMAAE overlap with beta cells and alpha cells, and the effect is worse than SCALEX. Therefore, we suggest that the hidden space of the IMAAE dimension should not be set too small.

IMAAE, MNN, scGen, iMAP, and SCALEX can all obtain corrected gene expression data. The difference is that the data obtained by MNN, SCALEX, and scGen contain negative numbers and cannot be directly used for differential expression analysis. In contrast, the output data of the IMAAE model conform to the real gene expression distribution, so it is convenient for differential expression analysis.

**Figure 9:**
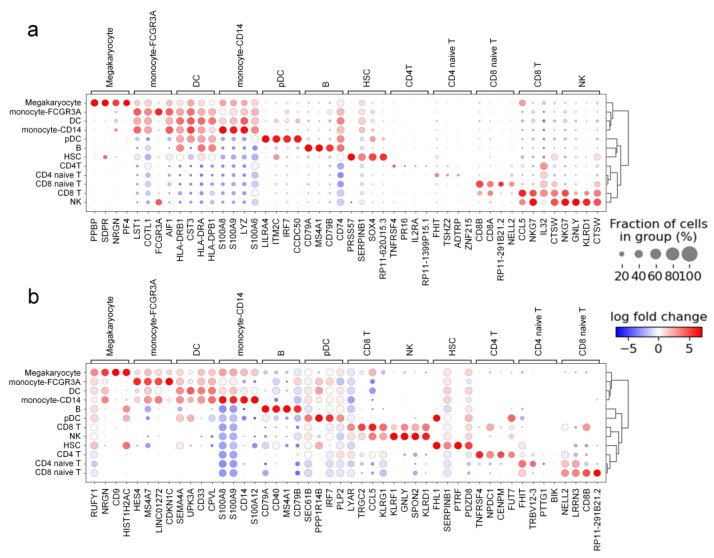
(**a**) Differentially expressed genes in the PBMC dataset before correction (**b**) Differentially expressed genes in the PBMC dataset after IMAAE correction.

We utilized the PBMC dataset to show the top four differentially expressed genes for each cell type before and after correction for batch effects (Figure 9). By comparison, we can find that the top four differentially expressed genes for each cell type changed before and after correction. The observed changes in the number and identification of the differentially expressed genes after correction are expected since the correction algorithm adjusts the data. This means that IMAAE, similar to the MNN algorithm, can uncover new findings for differential expression analysis.

### 3.5. Running Time Comparison

**Figure 10:**
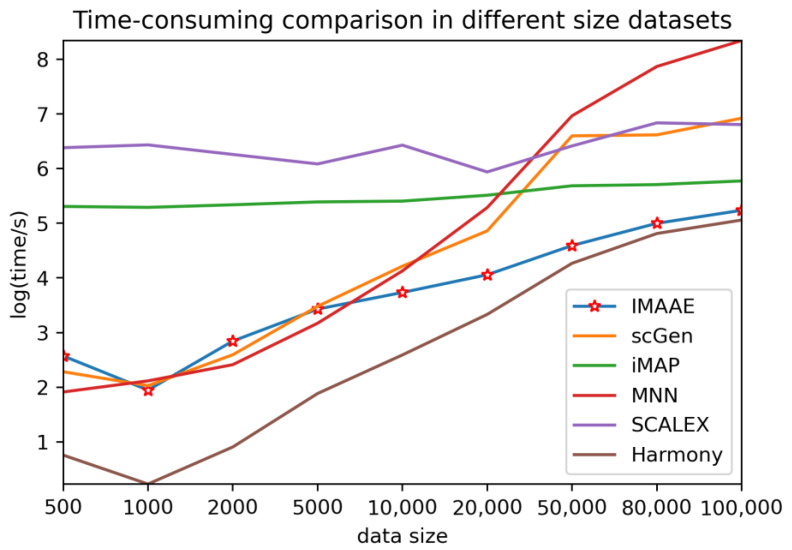
Comparison of the time performance of IMAAE with other methods on different-size simulation datasets.

At the same time, we also compare the time performance of each method on different-size simulation datasets (Figure 10). IMAAE adopts a downsampling strategy when constructing anchor batches, with a maximum of 1000 samples for each cell type, thus keeping the time overhead well under control. Compared with other methods, IMAAE achieves leading performance on large data sets.

### 3.6. Additional Experiment 1

**Figure 11:**
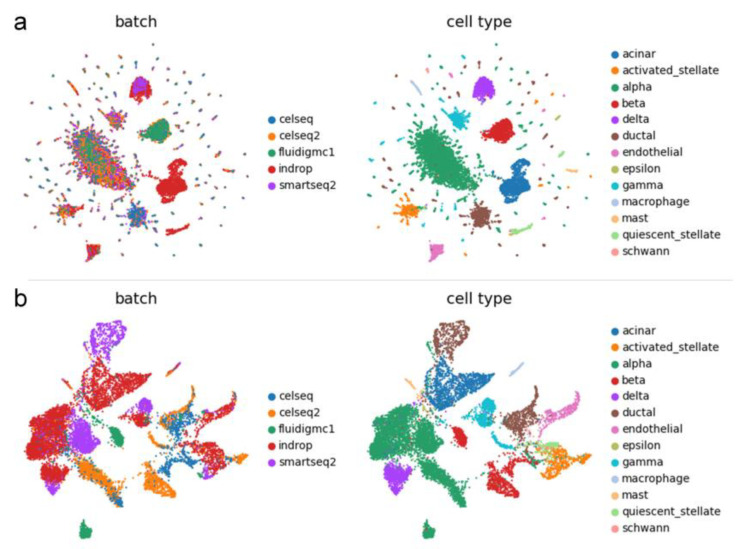
UMAP visualization of the data after AE and GAN correction on the Pancreas-subset dataset. (**a**) AE, (**b**) GAN.

At the beginning of the IMAAE model design, we found that autoencoder and generative adversarial networks have limitations in complex dataset scenarios. We use the Pancreas-subset for illustration. We first trained an autoencoder model, and the implementation results showed that it performs well on closed-set problems, while when dealing with partial-set problems, a lot of noise appears on the UMAP visualization graph if batches with fewer cell types are selected as anchors (Figure 11a), which means that the noise data is lack of constraints. We then trained a generative adversarial network model. The experimental results show that it works well on the two-batch problem, while when dealing with three batches and more, it produces undercorrection (Figure 11b), which indicates that it is difficult to fit the distribution of multiple high-dimensional data at the same time for the generative adversarial network [28]. Based on these results, we decided to use generative adversarial networks to constrain the hidden space of the self-encoder and created the IMAAE model. Based on the experimental results, we expect that the IMAAE model can be further applied in the field of multi-style data reduction and style migration.

### 3.7. Additional Experiment 2

**Figure 12:**
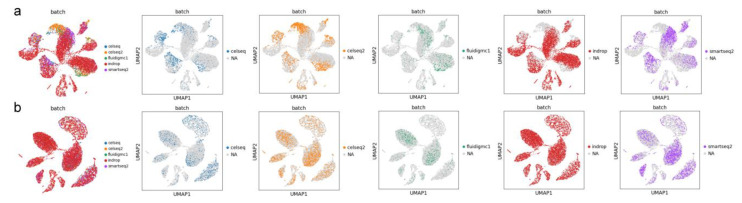
UMAP visualization of the distribution of each batch of the Pancreas dataset after IMAAE correction. (**a**) with steps 5 and 6, (**b**) without steps 5 and 6.

As depicted in Figure 12a, when applying steps 5 and 6, the corrected data of each batch still exhibit some batch-specific characteristics and show limited performance in batch mixing assessment. However, this data still retains the ability to further distinguish cell subtypes. In contrast, Figure 12b shows that without applying steps 5 and 6, the corrected data of each batch exhibit a more homogeneous distribution with no evident batch-specific characteristics, resulting in better batch mixing performance. Nevertheless, the data lose their ability to further subdivide cell subtypes. Researchers have the flexibility to decide whether to use steps 5 and 6 based on their task requirements.

### 3.8. Additional Experiment 3

**Figure 13:**
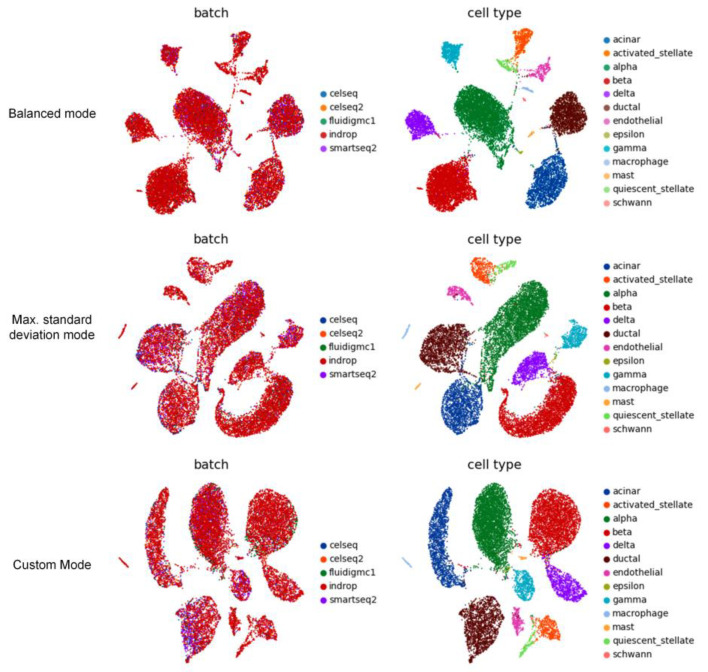
UMAP visualization of the Pancreas dataset corrected by IMAAE using three different anchor batch modes.

On the Pancreas dataset, we adopted three different approaches to establishing anchor batches. Among them, the anchor batch determined by the maximum standard deviation pattern is the “celseq” batch, and the anchor batch selected by the custom pattern is the “indrop” batch. Using IMAAE to correct batch effects, the UMAP visualization (Figure 13) shows that all three patterns can correct batch effects well, and it is difficult to distinguish which one is better. Quantitative evaluation (Table 2) shows that the balanced pattern performs better than the other two patterns, which may be because the anchor batches established by the balanced pattern are closer in spatial distance to each batch, while the anchor batches determined by the other two patterns are farther away from other batches. In practical applications, the pattern for establishing anchor batches should be selected flexibly according to the task requirements.

## 4. Conclusions

The batch effect poses a great challenge to scRNA-seq data analysis. In this study, we deeply analyze common dataset scenarios and propose the concepts of closed set, partial set, and open set, for which we design IMAAE, a deep learning-based supervised batch correction method. IMAAE is constructed by adversarial autoencoders to eliminate batch effects in scRNA-seq data by converting all batch cells to anchor batches.

One of the advantages of IMAAE over other methods is the flexibility to choose an anchor batch. Most of the current anchor-based methods select a batch with many cell types as the anchor and convert other batches to the anchor batch. At the same time, IMGG creates an intermediate batch and converts other batches to the intermediate batch. Our IMAAE combines the features of these two types of methods, allowing both the selection of a particular batch as an anchor batch and the creation of intermediate batches as anchor batches, providing more perspectives for downstream analysis.

Another advantage of IMAAE is that both corrected low-dimensional data and gene expression data can be obtained simultaneously. Thus, we can use the corrected gene expression data for differential expression analysis and the corrected low-dimensional data for some other tasks.

We must note that the reason why IMAAE achieves excellent performance is that it relies heavily on well-annotated datasets, so IMAAE is not a substitute for traditional unsupervised methods but compensates for the fact that labels cannot be fully utilized when cell types are known. In conclusion, we believe that IMAAE can be useful for single-cell analysis.

## Figures and Tables

**Table 1:** Quantitative assessment of batch effect correction methods using *ASW*, *ARI*, *NMI*, and corresponding *F*1 scores.

Data	Method	$ASW_{batch}$	$ASW_{celltype}$	$F1_{ASW}$	$ARI_{batch}$	$ARI_{celltype}$	$F1_{ARI}$	$NMI_{batch}$	$NMI_{celltype}$	$F1_{NMI}$
Pancreas	IMAAE	−0.1798	0.6930	0.8731	0.0091	0.9064	0.9468	0.0295	0.9183	0.9437
	scGen	−0.1293	0.3339	0.5154	−0.0019	0.7491	0.8572	0.0302	0.8359	0.8979
	MNN	−0.1055	0.1786	0.3075	0.0095	0.5468	0.7046	0.0310	0.7667	0.8561
	iMAP	−0.0630	0.2087	0.3488	−0.0015	0.8659	0.9288	0.0296	0.8259	0.8923
	SCALEX	−0.0442	0.2879	0.4514	0.0083	0.5870	0.7375	0.0182	0.7424	0.8455
PBMC	IMAAE	0.0084	0.3405	0.5069	0.0095	0.8172	0.8955	0.0059	0.8842	0.9359
	scGen	0.0092	0.3449	0.5116	0.0094	0.7031	0.8224	0.0059	0.8436	0.9127
	MNN	0.0140	0.1912	0.3203	0.0100	0.6280	0.7685	0.0063	0.7678	0.8662
	iMAP	0.0068	0.1732	0.2949	0.0098	0.5621	0.7171	0.0062	0.7321	0.8431
	SCALEX	0.0065	0.2385	0.3846	0.0085	0.5343	0.6944	0.0052	0.7285	0.8411
PBMC subset2	IMAAE	0.0094	0.4378	0.6072	0.0094	0.7946	0.8818	0.0059	0.8655	0.9253
	scGen	0.0180	0.4181	0.5865	0.0115	0.8094	0.8900	0.0053	0.8477	0.9153
	MNN	0.0154	0.1954	0.3261	0.0131	0.6796	0.8049	0.0061	0.7794	0.8737
	iMAP	0.0094	0.1466	0.2554	0.0148	0.5967	0.7432	0.0075	0.7022	0.8225
	SCALEX	0.0438	0.2075	0.3409	0.0060	0.6185	0.7625	0.0018	0.6810	0.8096

**Table 2:** Quantitative assessment of the correction results for three anchor batch models using *ASW*, *ARI*, *NMI*, and corresponding *F*1 scores.

Data	Method	$ASW_{batch}$	$ASW_{celltype}$	$F1_{ASW}$	$ARI_{batch}$	$ARI_{celltype}$	$F1_{ARI}$	$NMI_{batch}$	$NMI_{celltype}$	$F1_{NMI}$
Pancreas	IMAAE(Mean)	−0.1798	0.6930	0.8731	0.0091	0.9064	0.9468	0.0295	0.9183	0.9437
	IMAAE(Max. Std)	−0.1573	0.4617	0.6601	−0.0023	0.7794	0.8769	0.0302	0.8619	0.9127
	IMAAE(Custom)	−0.1618	0.6087	0.7989	0.0100	0.8988	0.9422	0.0294	0.8811	0.9237

## Data Availability

No new data were created or analyzed in this study. Data sharing is not applicable to this article.
